# Access to family planning for youth: perspectives of young family planning leaders from 40 countries

**DOI:** 10.12688/gatesopenres.13045.2

**Published:** 2019-11-11

**Authors:** Alice F. Cartwright, Jane Otai, Amelia Maytan-Joneydi, Courtney McGuire, Emily Sullivan, Adesola Olumide, Catherine Baye Easton, Ilene S. Speizer

**Affiliations:** 1 Department of Maternal and Child Health, Gillings School of Global Public Health, University of North Carolina at Chapel Hill, Chapel Hill, NC, 27599, USA; 2 Jhpiego, an affiliate of Johns Hopkins University, 1615 Thames Street, Baltimore, MD, 21231, USA; 3 Carolina Population Center, University of North Carolina at Chapel Hill, Chapel Hill, NC, 27516, USA; 4 Family Planning 2020, UN Foundation, 1750 Pennsylvania Avenue, Washington, D.C., 20006, USA; 5 Institute of Child Health, College of Medicine, University of Ibadan, Ibadan, Oyo State, Nigeria; 6 University College Hospital, Queen Elizabeth Road, Ibadan, Oyo State, Nigeria; 7 International Youth Alliance for Family Planning (IYAFP), 1750 Harvard Street NW, Washington, D.C., 20009, USA

**Keywords:** youth, adolescents, family planning, contraception, Africa, Asia

## Abstract

**Background**: With growing populations of young people, low and middle-income countries have renewed focus on reaching both unmarried and married youth with family planning (FP) services. Young people themselves bring an important perspective to guide future programmatic directions.

**Methods**: In October 2018, 207 youth leaders in FP from around the world completed an online survey prior to their participation at the International Conference on Family Planning (ICFP). These youth leaders provided their perspectives on the most important influencers for youth FP use, how easy or hard it is for youth to obtain FP, preferred sources of FP methods for youth, and perceptions of commonly used terms in FP programming. We examined differences in perceptions of unmarried and married youth’s access to and use of FP using bivariate analyses.

**Results**: Respondents reported that peers/friends were the most important influencer on use of FP among unmarried youth (80.2%), while spouse/partner was the most important for married youth (80.4%). Oral contraceptive pills, injectable contraception, and contraceptive implants were perceived as significantly harder for unmarried youth to access. Privacy, confidentiality, and anonymity were all important factors for the locations to access FP for unmarried youth, while married youth were more influenced by cost. None of the commonly used terms for FP were perceived positively by a majority of respondents, with the exception of ‘birth spacing’ by African respondents (51.0%).

**Conclusions**: These findings indicate that the preferences and needs of unmarried youth are different than married youth, but that all young people face barriers accessing FP. Unmarried youth seeking FP are more influenced by peers and friends and continue to face difficulty accessing methods compared to married youth. These findings indicate the importance of including youth perspectives in development of youth-focused family planning programs.

## Introduction

Across Africa, Asia, Latin America and the Caribbean, children under age 15 and youth aged 15–24 comprised 40–60% of the total population in 2017 ^1^. Many countries will see continued growth of their youth population throughout the next few decades ^2^. Adolescent pregnancy is a pressing global health challenge in these regions ^3, 4^. The risks of maternal and infant morbidity and mortality are high for young mothers and young women also suffer disproportionate consequences of unsafe abortion ^3^. Unintended pregnancies among young people may lead to them dropping out of school, reduce future employment opportunities, and increase the risk of poverty ^5^. Access to sexual and reproductive health services for youth is critical for their health and well-being and the overall successful achievement of goals laid out in the United Nations 2030 Agenda for Sustainable Development ^6^.

Many countries are actively developing strategies to expand family planning (FP) access for young people, as demonstrated by the fact that nearly all Family Planning 2020 (FP2020) commitment-making countries have a focus on adolescents and youth, including through provision of youth-friendly services, free contraceptives for adolescents, and ensuring consistent commodity supplies to youth-specific facilities ^7^. However, the level of detail of these commitments varies significantly. ‘Revitalized’ commitments in 2017 made by Ethiopia, Malawi, and Mozambique included plans to end child and early marriage, expand youth-friendly and school-based services, broaden method-mix availability, and include specific modern contraceptive use targets for unmarried sexually active adolescents ^8^.

With the growing number of young people, there is a renewed interest in determining which FP program strategies are most effective with this population. Prior reviews have demonstrated that there is no magic bullet for reaching young people with FP information and services ^9– 13^. Programs utilizing demand generation, engaging parents and community leaders, and training health care providers have been effective, but multi-component programming is needed ^9^. In addition, more evidence is needed on other approaches designed to reach young people, including providing services outside of health facilities, such as pharmacies and drug shops, determining how to reach the most vulnerable adolescent groups, and developing standardized definitions and indicators of what constitutes ‘youth-friendly’ services to strengthen implementation of this evidence-based strategy ^9, 10, 12^. The global community has undertaken FP programming with young people since the 1990s through large initiatives such as FOCUS on Young Adults (1995–2001) ^14^, YouthNet (2001–2006) ^15^, PRACHAR (2001–2013) ^16^, and, most recently, Adolescents 360 (2016-present) ^17^. However, there are still outstanding questions regarding the most effective ways to reach young people where and when they most need sexual and reproductive health and FP information and services. 

**Figure 1.**
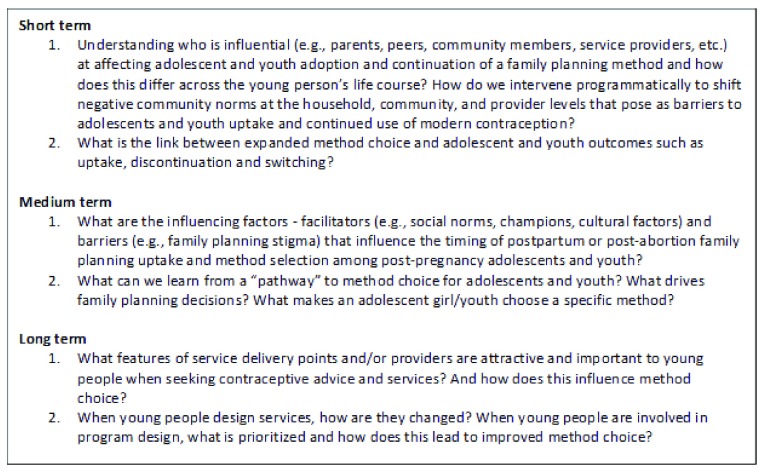
Top learning questions for expanded family planning method choice for youth prioritized by technical workshop attendees, 2018. Note: Learning agenda and meeting report available from here.

In March 2018, the Full Access, Full Choice project, in collaboration with FP2020, the Reproductive Health Unit of the World Health Organization, and the Evidence to Action project, convened a technical workshop of international organizations, United Nations agencies, and donors to identify key evidence and measurement needs to increase choice of and access to the full range of family planning methods and services for young people globally. The main output of the workshop was a global learning agenda of 41 questions. Participants ranked and identified the top two questions to be addressed in the short, medium, or long term ( Figure 1) ^18^.

An important step in addressing many of these learning agenda questions is gathering young people’s voices and inputs on their lived experiences to inform strengthened programs and policies that better meet their needs. With that in mind, this paper describes the results of a survey of youth FP leaders on a selection of the learning agenda questions to guide future FP priorities and programming for young people.

## Methods

### Survey instrument

A survey was developed by the Full Access, Full Choice project team at the University of North Carolina at Chapel Hill in collaboration with partners at Jhpiego, FP2020, and the International Youth Alliance for Family Planning. The survey questions were developed to align with a selection of the learning agenda questions from the collaborative technical workshop described above regarding influencers on youth FP use, how easy or hard it is for youth to obtain specific FP methods, where they most prefer to get each method, and why they prefer that source for the method. These questions were about attitudes and behaviors of unmarried and married youth in the respondents’ communities rather than questions about their own personal attitudes and behaviors. Respondents were also asked about their perception of commonly used FP terms, if they had suggestions for additional phrases that would be relevant to their community, and to provide, in their own words, suggestions for youth FP priorities in their communities. The survey included both closed and open-ended response options, depending on the question.

The survey was pilot tested with Masters students in a Master of Public Health (MPH) track at the University of Ibadan in Nigeria. The draft survey was shared with interested students by email to understand their comprehension of the questions, the length of time it took to complete the survey, two or three positive and negative elements of completing surveys in the past, and gather any other feedback. This confidential feedback was returned to their class representative and sent to the sixth author (AO), who then shared with all members of the study team to discuss. Selected questions were re-phrased based on this feedback and instructions added to some sections to improve clarity. Following the revisions from pilot testing, the finalized 45-question survey was programmed into the online survey platform Qualtrics. The survey was developed in English and French, the two main languages of target participants. A copy of the survey is included as *Extended data* ^19^.

### Target population and distribution of survey

The 2018 International Conference on Family Planning (ICFP) in Kigali, Rwanda was unique in that there was a multi-day pre-conference specifically for youth FP leaders, the target audience for the survey. In October 2018, six weeks before ICFP, the link to the online survey was sent by the youth conference organizers via email to 425 youth leaders in FP who planned to participate in the youth pre-conference. The survey was sent by the International Youth Alliance for Family Planning affiliates on behalf of the study team (contact information for participants was not shared with the study team). The youth leaders included young people identified by a video competition, young researchers whose research had been accepted for oral presentation at ICFP, youth leaders funded to attend the conference by family planning organizations, and winners of the 120 under 40 Champions of FP who were invited to participate in the youth pre-conference. The first survey question screened for eligibility and asked if respondents were between the ages of 18–35 years, the ages of participants at the youth pre-conference. If a respondent answered ‘no’, they were asked to confirm if they were below age 18 or above age 35. If their age fell outside of the eligible range, the survey ended. The survey was open for an approximate two-week period and one reminder email was sent out after the first week. Participants did not receive any incentive to complete the survey. The initial results were used to guide the development of a breakout discussion session at the youth pre-conference on adolescent and youth data use.

### Analysis

We first described the demographics of all eligible respondents who completed all questions in the survey, including their age, sex, geographic region of residence, urban/rural residence, marital status, and whether they were a student at the time of the survey, employed, or involved with activities related to family planning. Geographic region of residence was coded based on respondents’ report of their current country of residence. 

For the remaining analyses, we limited the sample to those respondents currently living in Africa, Asia, or Central/South America/Caribbean, as the sample living in Europe and North America was comparatively small (n=15) and the research questions were focused on youth in low and middle-income countries. We then examined what respondents reported were the main influencers of FP use for youth in their communities and compared the frequency by which each influencer group was mentioned for married and unmarried youth using paired t-tests. We then compared respondents’ perspectives on how easy or hard it is for youth to get specific methods and the main factors that affect where married or unmarried youth get a method and compared using chi-square tests. We also described respondents’ reactions to specific FP terminology by region using chi-square tests. All statistical analyses were conducted in Stata (v15.1, College Station, TX).

We identified reasons that respondents reported that unmarried and married youth most prefer particular sources for specific family planning methods through a thematic coding of open-ended responses conducted by the first and third authors (AFC and AMJ). After inter-coder reliability was established, the themes were summarized by whether the question referred to married or unmarried youth and by each FP method and demonstrative quotes were selected. Suggestions for other possible terms for family planning were also grouped thematically by the first and third authors and suggestions selected to represent unique suggestions, as well as geographic diversity. Finally, the second and eighth authors (JO and ISS) conducted a thematic analysis of the responses to an open-ended question asking respondents to describe in their own words what areas still need prioritization to increase youth access to FP. The themes were reviewed and summarized by the first author (AFC). Finally, when the data were prepared for open availability, respondents’ job titles were removed from the dataset to ensure their confidentiality.

### Ethical statement

The survey, consent statement, and protocol for data collection were submitted for review to the Institutional Review Board (IRB) at the University of North Carolina at Chapel Hill. The study was deemed exempt from IRB approval given that responses were anonymous and did not ask personal questions about the behaviors of the participants. Therefore, formal informed consent was not required. However, respondents were still informed at the beginning of the survey that their responses would be kept confidential and no names or identifying information linking them to the survey would be disclosed. In addition, they were told that it was their choice to complete the survey, their participation would not impact their participation in ICFP, and they were free to stop taking the survey at any time.

## Results

A total of 207 young people responded to and completed all the survey questions (49% response rate). Almost half of the respondents were aged 18–24 years (45.9%), with the remaining respondents aged 25–35 years. Approximately 60% of respondents identified as female ( Table 1). Almost three-quarters of respondents (72.4%) identified their current place of residence as a country in Africa, followed by 17.2% living in Asia and smaller proportions from other parts of the world (greater participation of African respondents is likely reflective of the fact that ICFP took place in Rwanda). Forty unique countries were represented. Three-quarters of participants were engaged in family planning related activities as part of their work or school. 

**Table 1.** Demographic characteristics of respondents.

Characteristic	Percentage of respondents (N=207)
Age (years)	
18–19	1.9
20–22	18.8
23–24	25.1
25–35	54.1
Sex ^†^	
Female	59.8
Male	39.7
Other (Gender non-conforming)	0.5
Region of Residence ^ǂ^	
Africa	72.4
Asia	17.2
Central/South America/Caribbean	3.0
Europe/North America	7.4
Residence ^ǂ^	
Urban (capital or other city)	66.0
Rural (town, village, or other rural area)	34.0
Married or in union ^§^	23.9
Current student ^§^	47.8
Employed ^§^	67.8
Engaged in activities related to family planning ^ǂ^	76.7

^†^ N=204; ^ǂ^ N=203; ^§^ N=205; ^ǂ^ N=206

Table 2 presents the main influencers that respondents reported affect the use of FP among unmarried or married youth in their communities. The main influencers reported for unmarried youth were peers and friends (80.2%) and boyfriend/girlfriend (65.2%), followed by media personalities/influencers (29.0%), community health workers (23.7%), and service providers (22.7%). Married respondents were significantly more likely to state that unmarried youth were influenced by their parents than unmarried respondents and those in Asia were significantly more likely to report media personalities/influencers impacting unmarried youth than African respondents (not shown). Reported influencers of married youth FP use were significantly different. Spouse or partner was reported as the most influential (80.4%), followed by service providers (44.1%), community health workers (39.2%), and peers/friends (36.3%). A similar question was asked about influencers of contraceptive choice and contraceptive continuation and the pattern of responses was similar (see *Underlying data*) ^19^.

**Table 2.** Main influencers of family planning use for unmarried and married youth ^†^.

	Unmarried (%, N=207)	Married (%, N=204)
Peers/friends ***	80.2	36.3
Boyfriend/girlfriend (unmarried) *** Spouse/partner (married)	65.2	80.4
Service providers ***	22.7	44.1
Community health workers ***	23.7	39.2
Media personalities/influencers ***	29.0	14.7
Parents *	15.0	23.0
Neighbors or others in community *	8.7	16.7
Aunts, uncles, other family ***	4.8	14.7
Religious leaders	5.8	6.9
Siblings	5.3	2.5
Teachers ***	8.7	1.0
Internet, social media, media *	2.4	0.5
Non-governmental organizations/ community-based organizations	1.0	0.5
Government leaders	0.0	1.0

†Respondents could provide up to three responses; *p<0.05, **p<0.01, ***p<0.001 comparing the percentage that gave each response for married versus unmarried youth.

**Figure 2.**
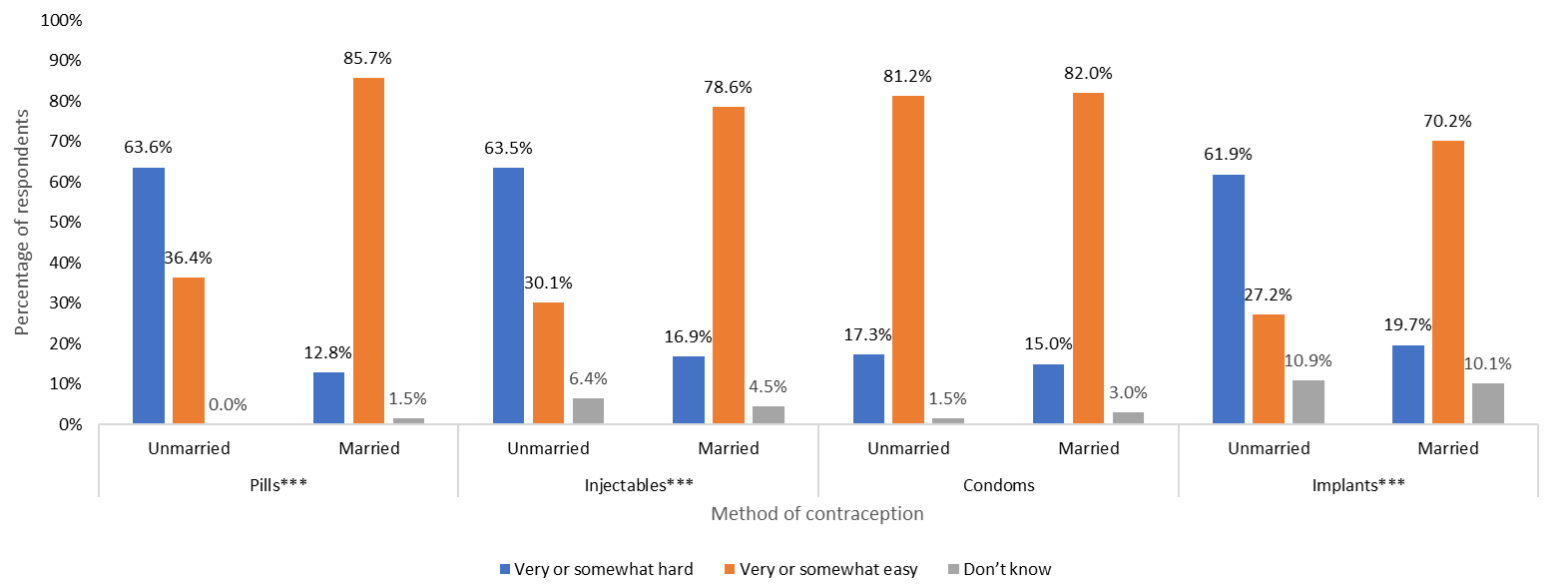
How easy or hard it is for unmarried and married youth to get family planning by method. ***p<0.001

Almost two-thirds of respondents said that it was very or somewhat hard for unmarried youth in their communities to access most FP methods (pills, injectables, and implants) besides condoms ( Figure 2). Conversely, 85.7% and 78.6% of respondents said it was very or somewhat easy for married youth to get pills and injectables (significantly different than unmarried youth). Asian respondents also felt it was significantly harder for unmarried youth to get injectables than African respondents, but were more likely to state that they did not know how hard it would be for unmarried youth to get implants (not shown). Further, while 70.2% reported that it was easy for married youth to get implants, 19.7% reported that implants are still somewhat or very hard for this group to get. Condoms were rated easy to get almost equally for unmarried and married youth.

**Figure 3.**
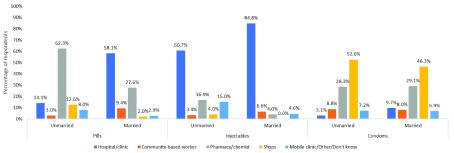
Main preferred source of family planning for unmarried and married youth by method.

Respondents also provided information about where unmarried and married youth in their communities most prefer to obtain different FP methods ( Figure 3). The preferred locations varied by method and marital status. According to respondents, unmarried youth most prefer to get pills from pharmacies and chemists (62.3%), while married youth most prefer to get their pills from a hospital or health clinic (58.1%). Respondents reported that both married (84.8%) and unmarried (60.7%) youth prefer hospitals or clinics to get injectables, though nearly one fifth of respondents said that unmarried youth would like to get injectables at a pharmacy/chemist. Married respondents were significantly more likely than unmarried respondents to think that unmarried youth would get pills or injectables from a hospital or health clinic. African respondents were also significantly more likely than Asian respondents to think that hospital or clinic would be the source for pills for married youth and injectables for unmarried youth, with Asian respondents citing community-based workers and pharmacies as a source of pills and community-based workers and mobile clinics more commonly for injectables (not shown). Shops were the most commonly preferred source for condoms for both unmarried and married youth, followed by a pharmacy/chemist for both groups. Other sources for condoms suggested by small numbers of respondents included vending machines/dispensers, youth clinics, peer educators, school/university, or at community events. Finally, respondents reported that both married (89.0%) and unmarried (80.7%) youth would most prefer to get implants at the hospital or health clinic (see *Underlying data*) ^19^.

Respondents were asked what factors have the most impact on where youth in their communities seek FP, as well as open ended questions about why they thought unmarried or married youth most preferred specific sources by method. Some different reasons emerged across methods and locations for married and unmarried youth. For example, in open ended responses, respondents reiterated that unmarried youth prefer to get pills at pharmacies/chemists due to privacy, confidentiality, and less judgement than they might expect to experience at a health facility, whereas respondents reported that married youth prefer to get pills at a hospital or clinic where they are free ( Table 3). Other reasons for preferring a particular source for a specific method included in Table 3 demonstrate the importance of access to and safety of injectables from ‘trained’ providers, as well as the perceived advantage of shops and pharmacies/chemists because they are discrete and there is more anonymity and less judgement passed during the business transaction of purchasing condoms. 

**Table 3.** Commonly reported reasons for most preferred sources of family planning for unmarried and married youth by method.

Method	Marital status	Location	Reasons	Sample quote
Pills	Unmarried	Pharmacy/chemist	Privacy, confidentiality	“ *People can't judge them easily, they may think that he/she* *went to bought [sic] other type of medicine*”
			No judgement, business transaction	“ *It is more of business such that no one can bring up moral* *issues*”
	Married	Hospital/clinic	Cost	*“The services are offered for free”*
			Marital status	*“Because they are married and the service providers will* *happily give them the service”*
Injectables	Both unmarried and married	Hospital/clinic	Safety, trained providers	*“Because they see [an] injection as something complex so* *they would rather prefer getting it from a professional”*
			Access, availability	*“The family planning clinics are [the] only places we can* *access injectables”*
Condoms	Both unmarried and married	Shops; pharmacy/ chemist	Easy access, convenience	“ *It's easy to buy from chemists, no prescription needed”*
			Discrete, anonymous	*“They find this more discrete because when leaving a* *pharmacy, no one will know why you went there” (translated* *from French)*
			No judgement, business transaction	*“You just walk up with money and you are given the product* *without questions or caution”* *“Chemist attendants do not query or lecture the unmarried* *people. It is just business”* *“Because at the shops they are not judged that they are* *using condoms as a couple since the society does not* *understand that married couples can use condoms”*

Respondents were then asked about how youth perceive commonly used terms in FP programming ( Table 4). The terms “birth spacing” (51.0%) and “contraception” (44.7%) were perceived most positively by African respondents. “Long acting and reversible contraception (LARC)” was perceived less positively, whereby 36% of African respondents reported that youth do not understand the terms and 32% reported that the term was viewed negatively. Almost two-thirds of Asian respondents reported that youth do not understand the term in their region. Married respondents were significantly more likely to report that youth do not understand the terms “LARC” or “birth spacing”, while unmarried respondents were more likely to state that youth felt negatively or neutral about “LARC” and positively about “birth spacing” (not shown). Respondents were asked to make suggestions for other possible terms for FP that might resonate with youth in their communities. A few selected responses in Table 5 demonstrate a desire for a more holistic discussion of the role of FP in the lives of youth, with many suggested terms including the removal of “family” from family planning in favor of “life” or “future” planning.

**Table 4.** Perceptions of commonly used FP terms by whether viewed by youth as positive, negative, or neutral by respondent region of residence ^†^.

	Positive	Negative	Neutral	Youth do not understand this term
**Family planning**				
Africa	37.9%	22.8%	24.8%	14.5%
Asia	38.2%	5.9%	35.3%	20.6%
**Contraception**				
Africa	44.7%	14.9%	28.4%	12.0%
Asia	36.4%	21.2%	27.3%	15.1%
**Long acting reversible contraception (LARC)**				
Africa	11.2%	31.5%	21.7%	35.6%
Asia	8.8%	17.6%	5.9%	67.7%
**Birth spacing** *				
Africa	51.0%	12.6%	21.7%	14.7%
Asia	26.5%	0.0%	41.2%	32.3%

†Central/South American/Caribbean (n=6) and Europe/North America (n=15) respondents not included due to small number of respondents

*p<0.05

**Table 5.** Suggestions for other possible terms for family planning.

Suggestion	Respondent country
“nyansapo: i.e. involving total discussions on matters of growth and maturation in all areas of life that enables one to disentangle complexities of life with wisdom, skill, dexterity and profound capacities to adapt to the exigencies of life including planning the life of family in relation to one's aspirations of 'family' that has the wherewithal to make choices freely and with support availability where needed”	Ghana
“Goal Keeper-When you use FP it’s like you keep babies from coming out.”	Malawi
“future protection- this term is normally used by adolescent to mean protecting future by contraception”	Kenya
“Future Plan”	Kenya
“Life planning”	Nigeria
“For many people family planning implies that you already have a family, and it excludes unmarried people”	Guatemala

Finally, respondents were asked what areas need more prioritization to increase youth access to FP in their own words. The key themes focused on education, reducing stigma, and engaging youth. Respondents suggested that sex education should be made available throughout the school setting from high school through college. Others suggested that it was key for policy makers, programs, and providers to reach youth ‘where they are’, including making information and methods available online, through social media platforms and apps, and at places where youth gather, like clubs and colleges/universities. Respondents also believed that stigma and myths related to FP still need to be addressed, through education of community and religious leaders and support for parents to have open conversations about sex and FP with their children. Others suggested reframing FP as more holistic ‘life planning’.

Respondents said that stigma can be reduced by ensuring that FP is integrated into national health care programs, removing requirements for minors to get parental permission to access FP, and removing bans on advertising for condoms or FP on television or radio. Young people indicated that youth should be engaged at all levels of planning in order to make programs and policies that are more responsive to their needs. Other suggestions included identifying ‘younger’ providers to serve youth and increasing hours of FP clinics to make them more convenient for students. Finally, respondents suggested prioritizing high quality and reliable data collection on the perceptions and experiences of young people related to these issues and building the capacity of local organizations to ensure sustainable advocacy.

## Discussion

This survey of young FP leaders gives insight into several factors influencing youth FP use. A key point was the extent to which respondents noted that FP use for unmarried youth is differentially influenced by those they are interacting with the most: their peers and friends and boyfriends/girlfriends, but also media personalities or influencers and internet and social media. As access to internet-enabled mobile phones continues to grow, efforts to reach youth with health messages have begun to shift to mobile phones and social media, with the goal, as respondents noted, of meeting them ‘where they are’ ^20^. The challenge that remains is determining the best way to reach specific groups of youth, as access to mobile phones may be disproportionate across countries in urban vs. rural settings and by socioeconomic status. While many FP mobile health (mHealth) interventions to date have focused on tools to help providers with counseling ^21– 23^, products such as Nivi, a digital marketplace for information, recommendations, and referrals for FP, could offer key opportunities to target unmarried youth with accurate information, answers to questions, and linkages to care.

Stigma related to unmarried youth’s access to FP continues to persist and is reflected by most respondents reporting that it is very or somewhat hard for unmarried youth to access pills, injectables, or implants. While expanded youth-friendly services is a stated goal by many countries, these efforts should make sure to prioritize the specific factors that respondents characterized in preferred sources: affordability, privacy/confidentiality/anonymity, discrete staff, no judgement, and staff who make clients feel comfortable. As respondents suggested, there is still work to be done to reduce stigma and provider bias in communities and health facilities. It is important that programs targeting young people do not simply train providers on youth-friendly services, but also include other individuals who interact with young people (e.g. pharmacists, teachers, and religious leaders). This will ensure that young people are surrounded by supportive adults to help facilitate their access to FP services where and when they need it. Another option to meet the desire for privacy/confidentiality and reduced stigma may include increasing access to additional methods at pharmacies/chemists and drug shops, such as subcutaneous Depo Provera (DMPA-SC), which young people could purchase and administer themselves at home or another private location ^24, 25^.

Language was also an important focus for feedback in this survey. None of the most commonly used programmatic terms for FP were viewed particularly positively by young people, except for ‘birth spacing’ in Africa. While there may be numerous existing local translations and slang for different contraceptive methods, respondents emphasized that many of the commonly used phrases do not necessarily resonate with youth. Suggestions for different terminology focused on ‘future’ planning or ‘life’ planning, with the use of ‘family’ possibly alienating unmarried youth. These findings are in line with those of recent programs, including Adolescents 360, which has shifted their initial focus on increasing contraceptive use to an approach which supports the development of young women’s financial and entrepreneurial skills, with contraceptive use framed as a resource to help them achieve more immediate life goals ^26^. Programmatic efforts may want to focus on developing alternate, context-specific terms for FP, contraception, and LARC that resonate more closely with the lived experiences of young people in their settings. Some other programmatic examples to date include the branding associated with Diva Centres in Zambia and Future Fab in Kenya (both collaborative projects of Marie Stopes and IDEO.org) ^27, 28^.

## Limitations

This study has some important limitations. Most of the respondents were based in Africa, likely because the ICFP took place in Rwanda and those from outside Africa may have been less able to travel to and attend the meeting. Therefore, responses presented are likely more applicable to African country-contexts than other parts of the world, particularly as the types of FP methods and facilities at which they are available may vary in other contexts. However, stigma toward sexually active unmarried youth using FP exists across many countries, so it is possible that similar conclusions would be reached with a more geographically diverse sample of respondents. In addition, this study focused exclusively on respondents who were engaged with ICFP and thus is not generalizable of all young people. In addition, we are unable to determine sociodemographic differences between those who completed the survey and those who did not and there may be important differences. Since the survey was sent to people planning to attend the ICFP youth pre-conference, it is likely that all of those to whom the survey was distributed have participated in programmatic efforts directed at youth in their own countries and respondents likely felt comfortable speaking about the experiences of their communities and peers. However, we recognize that respondents' general impressions about youth may not capture the full breadth of experiences of all youth in their country and these experiences likely vary based on various characteristics including gender, race/ethnicity, or religion. This is evidenced by some discrepancies in married and unmarried respondents’ impressions of unmarried youth, for example.

## Conclusions

This paper provides direct feedback from youth engaged in FP issues within their communities. Their perspectives are particularly useful as governments, policy makers and program planners seek to increase equitable access to FP for youth in their countries and operationalize their FP2020 commitments related to adolescents and youth. As reflected here, to truly reach young people, ‘youth-friendly’ services must continue to focus on meeting youth where they are, reducing stigma in communities and bias among providers, using language and programs that integrate FP into larger issues of achieving healthy lives and futures, and finally, continuously engaging a youth perspective on the success of these efforts.

## Data availability

### Underlying data

Harvard Dataverse: Access to Family Planning for Youth: Perspectives of Young Family Planning Leaders from 40 Countries. https://doi.org/10.7910/DVN/M1OHTP ^19^

This project contains the following underlying data:

- ICFP Youth Survey Data 2019 06 21.tab (Raw data from all survey respondents and additional variables created for analysis)

### Extended data

Harvard Dataverse: Access to Family Planning for Youth: Perspectives of Young Family Planning Leaders from 40 Countries. https://doi.org/10.7910/DVN/M1OHTP ^19^

This project contains the following extended data:

- Additional data documentation ICFP Youth survey 2019 06 21-1.pdf (Documentation describing additional variables created for data analysis)
- Survey_of_ICFP_Youth_Pre-Conference_Participants 2019 06 21.pdf (Survey of ICFP youth conference participants in English)
- Survey_of_ICFP_Youth_Pre-Conference_Participants_FR 2019 06 21.pdf (Survey of ICFP youth conference participants in French)

Data are available under the terms of the Creative Commons Zero "No rights reserved" data waiver (CC0 1.0 Public domain dedication).
