# Supplementary material for: Access to family planning for youth: perspectives of young family planning leaders from 40 countries
Source: Gates Open Res. 2019 Nov 11;3:1513. Originally published 2019 Jul 25. [Version 2] doi: 10.12688/gatesopenres.13045.2 (PMC6978846; doi:10.12688/gatesopenres.13045.2)
Supplement: Peer review history

# Peer review: Reviewer response for version 2

**DOI:** 10.21956/gatesopenres.14230.r28441

**Published:** 2020-02-19

**Authors:** Venkatraman Chandra-Mouli

**Affiliations:** 1 Department of Reproductive Health and Research, Human Reproduction Programme, World Health Organization (WHO), Geneva, Switzerland

Overall comments:

 My two main comments are in the use of the term youth, which is widely used and understood to include individuals in the 15-24 year age group to cover a much wider age group, without any mention of this in the title or the abstract, and that data is not presented disaggregated by age. (The reality of 15 year olds is very different, as the authors are well aware from that of 35 year olds).

 Another comment is that it is not clear from the methods section whether emergency contraception was mentioned or not. It is not referred to at all in the paper.

 The paper has been reviewed by Mara Decker and Andrea Hoopes. I have gone over their comments and the responses to them.  I have not covered the issues they have.

 Specific comments:

 Pages 1-2:

 25-35 is above the UN definition of young people. This is not a problem in itself; one can survey any age group one wants to. But when the term young people is used, it is widely understood to conform with the UN definition of 10-24. There is no mention of this extended age band either in the title or the abstract.  

 Page 3:

 Introduction

 No comments.

 Methods:

 No comments.

 Page 4:

 Methods continued:

 Right hand column, para 1, line 1: 25-35 is above the UN definition of young people. This is not a problem in itself; one can survey any age group. But when the term young people is used, it is widely understood to conform with the UN definition of 10-24. Given that there is no mention of the extended age band either in the title or the abstract, this could be misleading. 

 Page 5:

 From table 1, it is evident that over 2/3 of the respondents are employed and ¾ in the FP field. This is reflected in the title, but the point that this group are unlikely to be representative of the wider community of young people must be stressed.

 Page 6:

 In figure 2, pills, injectables, condoms and implants are mentioned. Did the survey tool list these methods or was the feedback spontaneous? If the survey tool listed the methods, was emergency contraception part of the list? If it was not listed, did it not come up spontaneously? Was abortion mentioned at all in the responses?

 Page 7:

 No suggested changes.

 Page 8:

 No suggested changes.

 Page 9:

 No suggested changes.

 Page 10:

 No suggested changes.

I confirm that I have read this submission and believe that I have an appropriate level of expertise to confirm that it is of an acceptable scientific standard.

# Peer review: Reviewer response for version 2

**DOI:** 10.21956/gatesopenres.14230.r28412

**Published:** 2020-02-18

**Authors:** Claire D. Brindis

**Affiliations:** 1 Philip R. Lee Institute for Health Policy Studies, University of California, San Francisco (UCSF), San Francisco, CA, USA; 2 Bixby Center for Global Reproductive Health, San Francisco, CA, USA

This article helps to continue to demonstrate the existing challenges to assure access to contraceptive care among adolescents. It would be helpful if authors provided more information on those who were not responding to better understand potential bias; more information on how the study aimed at assuring that respondents actually did not answer to reflect their own experience vs. the experience of those they represented (Page 3), and how other factors, such as their own length of service in the field (younger respondents vs. older; gender and other profile factors from the sample to further "unpack" the results. In other words, what was the profile of non-respondents---even basic information. A 49% response rate is acceptable in such studies, but would be helpful to "defend" that this is representative enough. Also, it was not clear whether individuals could provide more than one answer regarding their main influencers and whether individuals who noted more than 1 vs 2-++ had different types of answers. on page 6, paragraph before Discussion, helpful to differentiate between marital status and also younger vs. older respondents. Under Discussion, helpful to still acknowledge and work with parents, peer family members, such as older siblings, and aunts/uncles as helpful in supporting young people. Discussion would also benefit re: other barriers such as financial, transportation, confidentiality in these countries as these factors remain major challenges.

I confirm that I have read this submission and believe that I have an appropriate level of expertise to confirm that it is of an acceptable scientific standard.

# Peer review: Reviewer response for version 2

**DOI:** 10.21956/gatesopenres.14230.r28440

**Published:** 2020-02-10

**Authors:** Anastasia Gage

**Affiliations:** 1 Department of Global Community Health and Behavioral Sciences, School of Public Health and Tropical Medicine, Tulane University, New Orleans, LA, USA

Young people’s access to safe, voluntary family planning is a human right and an important programmatic and policy issue. This survey report is based on a unique data set of young family planning leaders who attended the 2018 International Conference of Family Planning.

 The contribution of the report to existing knowledge about young people’s access to family planning has not been articulated by the authors. This is important because the report is descriptive and, though quantitative, it is based on a small non-representative sample of 207 young conference participants from 40 countries. As the average number of respondents per country is approximately 5.2, it would be difficult to justify making policy and programmatic decisions on such a small sample size of participants, given the existence of large-scale data sets from Performance Monitoring and Accountability 2020 and the Demographic and Health Surveys.

 Therefore, the authors should highlight what makes their study unique and what contributions the report makes to what is known about young people’s access to family planning services. The overall importance of the study could be supported by a brief description of the strengths and weaknesses of existing studies, a comparison of the findings of those studies with those of the present report, and an identification of research and programmatic gaps that the study has contributed to filling.

 The main limitation of the study, as a previous reviewer points out, is that the perspectives of the participants may be biased if they do not “share common identities or lived experiences related to gender, race/ethnicity, disability status, language,” etc. with other young people in their communities. The authors do include a statement in the discussion section that the study is not generalizable to all young people but do not expand on the full extent of the bias. We do not have much information about the socioeconomic background of the youth family planning leaders except that 48% are current students. I would encourage the authors to present data on the level of education of study participants, if available.

I confirm that I have read this submission and believe that I have an appropriate level of expertise to confirm that it is of an acceptable scientific standard, however I have significant reservations, as outlined above.

# Peer review: Reviewer response for version 2

**DOI:** 10.21956/gatesopenres.14230.r28216

**Published:** 2020-01-22

**Authors:** Mara Decker

**Affiliations:** 1 Institute for Health Policy Studies, University of California, San Francisco, San Francisco, CA, USA

The authors added further clarification of many of the items identified in the first review. Note that the abstract still reads as if the "young people" interviewed are reporting on their own perceived barriers. 

 This paper is sufficiently detailed for indexing.

I confirm that I have read this submission and believe that I have an appropriate level of expertise to confirm that it is of an acceptable scientific standard.

# Peer review: Reviewer response for version 2

**DOI:** 10.21956/gatesopenres.14230.r28217

**Published:** 2019-11-18

**Authors:** Andrea J. Hoopes

**Affiliations:** 1 Adolescent Center, Kaiser Permanente Washington, Seattle, WA, USA

The amended version has clarified the questions I raised in my initial review and is suitable for indexing.

I confirm that I have read this submission and believe that I have an appropriate level of expertise to confirm that it is of an acceptable scientific standard.

# Peer review: Reviewer response for version 1

**DOI:** 10.21956/gatesopenres.14165.r27720

**Published:** 2019-09-11

**Authors:** Mara Decker

**Affiliations:** 1 Institute for Health Policy Studies, University of California, San Francisco, San Francisco, CA, USA

This is an innovative paper that capitalizes on surveying youth leaders who were planning to attend the 2018 ICFP Conference in Rwanda. I appreciated the efforts to incorporate both qualitative and quantitative data and analysis. The topics they attempt to cover, including perceived barriers to FP by contraceptive type and marital status, are important to consider while making programming and policy decisions.

 One issue when reading the manuscript is understanding exactly how the questions were asked. When I first read it, I assumed the respondents were answering for themselves. I had to read it a second time before realizing they were responding on behalf of “youth”. It appears that they were given the instruction to consider adolescents and youth “in the community you currently reside.” That contextualization is critical to determine the relevance of the responses. (Similarly, did older, married respondents have different answers from younger, unmarried respondents?)

 The paper needs to stress throughout the methods and results section that these are not individuals’ personal responses to their own barriers or influencers, but rather what they think other youth may perceive. You allude to this in the limitations, but may want to further highlight that these responses are “one-step removed.”

 Another issue in the framing (including the title) is that this is a global survey of youth, when in fact the vast majority were from Africa. I suggest reanalyzing the data in Table 2-3 and Figures 2-3 to see if there are variations by location. Even if you don’t include all that information in the tables themselves, you could add a sentence or two to the text stating if variations by location were found.

 Two other minor points:

 In the introduction, please consider if all the acronyms are necessary. If you only use a term a couple times, you can just keep the complete wording. Also, if you do include an acronym (such as FP2020), you only need to define it once.

 Finally, when asking about commonly used FP terms, did you include sexual and reproductive health? Did anyone mention that? You have the term in the first paragraph of the introduction, but it isn’t used again. (In my experience, youth understand that much more easily than FP.)

I confirm that I have read this submission and believe that I have an appropriate level of expertise to confirm that it is of an acceptable scientific standard, however I have significant reservations, as outlined above.

# Response

**Published:** 2019-11-02

**Authors:** Alice Cartwright

Thank you to the reviewer for their comments. We think it is important to make the distinction that those who are not a part of the group about which they are being asked (e.g. asking married people about unmarried youth) may have different impressions. These additional results (where significant) have been added to the Results section.

 Thank you to the reviewer for their suggestion to underscore that respondents to the survey were providing their impressions of influencers on and access to FP for youth in their communities (not for themselves). We have added more clarification throughout the Methods and Results sections to underscore that respondents are giving their impression on behalf of youth in general. We have also added additional detail to the Limitations section to clarify that respondents were asked to provide their impressions of youth in their communities, and these impressions are likely not applicable to all youth in their countries, especially considering the variety of race/ethnicity/tribal and religious identities that may exist.

 We have added in the text places where significant regional differences in responses were identified.

 We have removed acronyms only mentioned once or twice and confirmed that the acronyms used frequently in the paper, including FP2020 and ICFP are only defined once (with an additional explanation in the abstract if necessary).  

 In this survey, we asked respondents “Do adolescents/youth in your community perceive the following terms positive, negative, or are they neutral about them?” and then listed the terms “family planning”, “contraception”, “long acting and reversible contraception (LARC)”, and “birth spacing”. We then asked respondents “Are there more appropriate words/phrases for these terms that are preferred in your community? If yes, please provides these other terms and their definitions.” It was from these responses that we extracted unique and thematic responses, which are presented in Table 5.

# Peer review: Reviewer response for version 1

**DOI:** 10.21956/gatesopenres.14165.r27714

**Published:** 2019-09-03

**Authors:** Andrea J. Hoopes

**Affiliations:** 1 Adolescent Center, Kaiser Permanente Washington, Seattle, WA, USA

The authors present findings of a descriptive, survey-based study using a convenience sample of attendees at the International Conference on Family Planning. This study provides some valuable insights applicable to individuals and programs involved in improving the sexual and reproductive health of young people. Many of the findings are echoed in previous studies and reports of what barriers young people face in accessing FP services.  A key finding that is less well understood is how branding and language may influence willingness to engage in services. I recommend that the authors contextualize these findings in terms of what else is known about language/branding of services. 

 The authors' goal to meaningfully engage youth in this effort is appreciated. In the limitations, the authors point out that these data may not be representative of youth in all LMICs, and this is an important point. It is also necessary to point out that assumptions respondents are making about others in the community may have biases, particularly if those individuals do not share common identities or lived experiences related to gender, race/ethnicity, disability status, language spoke, religion, etc. Furthermore, the findings are subject to selection bias based on who chose to complete the survey. Were there any meaningful differences between respondents and non-respondents? Is this data available?

 A few additional comments:

 Please explain why you included individuals up to age 35 as this does not fit within the definition of youth or young people. 

 Did participants receive any incentives to complete the survey?

 Please consider expanding further upon Table 5 - are there any existing efforts to integrate these suggestions (like holistic life planning efforts) into youth FP services?  As is, this table is somewhat difficult to interpret or understand the implications. 

I confirm that I have read this submission and believe that I have an appropriate level of expertise to confirm that it is of an acceptable scientific standard, however I have significant reservations, as outlined above.

# Response

**Published:** 2019-11-02

**Authors:** Alice Cartwright

The reviewer makes an important point that respondents are making generalizations for ‘youth’ in their communities and not making specific distinctions of the needs or preferences of more specific demographic groups. We have more clearly made the distinction in the paper that the questions regarding influences and preferences regarding terminology are phrased to refer to married/unmarried ‘adolescents and youth’, while the questions about access to contraception are phrased differentially regarding married/unmarried ‘people’ for access to pills and condoms or married/unmarried ‘young women’ for injectable contraception and implants.

 Unfortunately, respondents were asked for their demographic information at the end of the survey, and therefore we do not have the ability to make conclusions about differences between respondents and non-respondents. We have added a mention of this in the Limitations section, as well as an acknowledgement that statements about ‘youth’ generally may not capture the diversity of experiences among all identities.

 While generally accepted definitions, including that of the World Health Organization, are of youth as ages 15-24 years and young people as 10-24 years, some countries’ definitions of youth are not always consistent with these ranges. For example, while the Nigerian Federal Ministry of Health aligns with the WHO definition, the Federal Ministry of Youth does not. At  the time of the study, the definition of youth used in the National Youth Policy in Nigeria (2009) was specified as all males and females aged 18 – 35 years who are citizens of the Federal Republic of Nigeria. The invitees to the ICFP Youth Pre-Conference included those up to age 35. We have therefore presented results from the full range of respondents, as those 15-24 only included 46% of total respondents and those in the 25-35 age range can provide interesting insights into their younger peers.

 Respondents did not receive any incentives to complete the survey. This detail has been added to the Methods section.

 We have added additional text within the Results section to better contextualize the feedback from respondents (i.e. moving away from the emphasis on “family” in family planning). We have also added information on programmatic efforts by Adolescents 360 in Ethiopia, Tanzania, and Nigeria that have supported young women in entrepreneurial and financial skills, with contraceptive use presented as a tool to help them achieve other goals.
